# Characterisation of the Immunophenotype of Dogs with Primary Immune-Mediated Haemolytic Anaemia

**DOI:** 10.1371/journal.pone.0168296

**Published:** 2016-12-12

**Authors:** James W. Swann, Kelly Woods, Ying Wu, Barbara Glanemann, Oliver A. Garden

**Affiliations:** 1 Department of Clinical Science and Services, Royal Veterinary College, Hawskhead Lane, North Mymms, Hatfield, Hertfordshire, United Kingdom; 2 Immune Regulation Laboratory, Royal Veterinary College, London, United Kingdom; Colorado State University, UNITED STATES

## Abstract

**Background:**

Immune-mediated haemolytic anaemia (IMHA) is reported to be the most common autoimmune disease of dogs, resulting in significant morbidity and mortality in affected animals. Haemolysis is caused by the action of autoantibodies, but the immunological changes that result in their production have not been elucidated.

**Aims:**

To investigate the frequency of regulatory T cells (Tregs) and other lymphocyte subsets and to measure serum concentrations of cytokines and peripheral blood mononuclear cell expression of cytokine genes in dogs with IMHA, healthy dogs and dogs with inflammatory diseases.

**Animals:**

19 dogs with primary IMHA, 22 dogs with inflammatory diseases and 32 healthy control dogs.

**Methods:**

Residual EDTA-anti-coagulated blood samples were stained with fluorophore-conjugated monoclonal antibodies and analysed by flow cytometry to identify Tregs and other lymphocyte subsets. Total RNA was also extracted from peripheral blood mononuclear cells to investigate cytokine gene expression, and concentrations of serum cytokines (interleukins 2, 6 10, CXCL-8 and tumour necrosis factor α) were measured using enhanced chemiluminescent assays. Principal component analysis was used to investigate latent variables that might explain variability in the entire dataset.

**Results:**

There was no difference in the frequency or absolute numbers of Tregs among groups, nor in the proportions of other lymphocyte subsets. The concentrations of pro-inflammatory cytokines were greater in dogs with IMHA compared to healthy controls, but the concentration of IL-10 and the expression of cytokine genes did not differ between groups. Principal component analysis identified four components that explained the majority of the variability in the dataset, which seemed to correspond to different aspects of the immune response.

**Conclusions:**

The immunophenotype of dogs with IMHA differed from that of dogs with inflammatory diseases and from healthy control dogs; some of these changes could suggest abnormalities in peripheral tolerance that permit development of autoimmune disease. The frequency of Tregs did not differ between groups, suggesting that deficiency in the number of these cells is not responsible for development of IMHA.

## Introduction

Immune-mediated haemolytic anaemia (IMHA) is considered to be the most common autoimmune disease of dogs and typically results in severe anaemia that develops acutely [1]. Development of IMHA in dogs is related to production of antibodies specific for normal molecules on the surface of erythrocytes. These antibodies cause activation of the complement cascade, resulting in intravascular lysis of red blood cells, or they opsonise red blood cells to facilitate phagocytosis by cells of the monocyte phagocyte system (MPS) in the liver and spleen [2]. Previous studies of dogs with IMHA indicate that autoantibodies and autoreactive T cells are most commonly specific for epitopes on glycophorin molecules expressed on the surface of red blood cells, but patterns of reactivity are not consistent among individual dogs [3,4].

Production of autoantibodies indicates failure of peripheral immune tolerance, a complex network of humoral and cellular factors that normally prevent T cells emigrating from the thymus from mounting responses against normal host tissues [5]. Regulatory T cells (Tregs) derived from the thymus represent one of the factors that contributes to maintenance of peripheral tolerance: these cells are recognised in mice, people and various domestic species, including dogs, by expression of the transcription factor FoxP3, expression of CD4 and constitutive display of the interleukin(IL)-2 receptor alpha chain (CD25) [6–8]. Regulatory T cells have the ability to suppress the activity of conventional T cells, B cells and cells of the innate immune system by various mechanisms, including the production of cytokines (such as IL-10 and transforming growth factor beta, TGFβ), surface expression of inhibitory molecules (such as cytotoxic T lymphocyte antigen (CTLA)-4) [9], and direct lysis or metabolic disruption of target cells [6].

Soluble signalling molecules, including cytokines, are also likely to contribute to maintenance of peripheral tolerance but their exact role has proved difficult to define because their effects are dependent on immune context. These cytokines also permit integration of Treg activity with other components of the immune system that have regulatory activity. Overproduction of the pro-inflammatory cytokines IL-6 and tumour necrosis factor alpha (TNFα) has been associated with disease activity in models of human autoimmune disease; this could be related to their ability to decrease the suppressive activity of Tregs [10,11]. Conversely, IL-2 and 10 are required to maintain the size and activity of the normal Treg compartment in mice [12,13], such that animals with experimental deficiencies in either molecule develop spontaneous autoimmune diseases [14,15].

Greater understanding of abnormalities in peripheral immune tolerance in dogs with IMHA may guide future attempts to produce therapeutic agents that might control the autoimmune response [16]. This approach has been fructiferous in people, in whom recombinant monoclonal antibodies against TNFα and CD20 (a molecule exclusively expressed on the surface of B cells) are employed in treatment of warm autoimmune haemolytic anaemia (wAIHA) [17], a disease that closely resembles IMHA in dogs.

In this project, we hypothesised that the immune phenotype of dogs with IMHA would differ from that of healthy dogs or dogs with inflammatory diseases without autoimmune aetiology, revealing defects in peripheral immune tolerance in the former group. Specifically, we hypothesised that dogs with IMHA would have a decreased frequency of circulating Tregs and lower serum concentrations of IL-10 but higher concentrations of pro-inflammatory cytokines than the control dogs in other groups.

## Materials and Methods

### Identification of cases and collection of samples

Three groups of dogs were recruited to the study over a period of 28 months, from July 2013 to January 2016: healthy control dogs (HC), dogs with inflammatory diseases (INF) and dogs with primary IMHA (IMHA). Samples were collected from healthy dogs belonging to staff and students at the study institution under license from the Animals (Scientific Procedures) Act 1986. None of these dogs had a history of autoimmune disease; physical examination was unremarkable in all cases.

Dogs in the INF group were recruited after they were diagnosed with an inflammatory disease that was not considered to have an immune-mediated aetiology. None of these dogs had a history of previous autoimmune disease, and all were followed until the conclusion of the study (in January 2016) to ensure that their clinical signs were not ultimately ascribed to autoimmune disease. All dogs in this group underwent diagnostic testing, including a complete blood cell count (CBC), serum biochemical profile and imaging of the thorax and abdomen, with additional tests selected by the attending veterinarian as considered appropriate. Dogs were excluded from this group if they were diagnosed with or had a previous history of autoimmune disease, or if a likely diagnosis could not be established.

Dogs were diagnosed with IMHA if they were anaemic (with a packed cell volume of less than 35%) and fulfilled at least one of the following criteria that suggested the presence of antibodies specific for erythrocytes: persistent microscopic or macroscopic agglutination of blood after dilution in saline, titre greater than or equal to 1:16 in a direct anti-globulin test (DAT, Coombs’ test), or presence of spherocytes on assessment of a fresh blood smear by a board certified veterinary clinical pathologist. Agglutination of blood was assessed by mixing one drop of EDTA-anticoagulated blood with one drop of 0.9% saline on a glass slide before macroscopic and microscopic inspection. Dogs were excluded from this group if they had previously been diagnosed with IMHA (and were suffering a relapse of the same disease), or if any underlying cause of immune-mediated disease was suspected from perusal of the clinical history, physical examination findings or results of the CBC, serum biochemical profile, serological tests for vector-borne infectious agents, imaging of the thorax and abdomen, and any other tests that were considered appropriate by the attending clinician. Dogs were also excluded if they had received immunosuppressive drugs prior to presentation at the study institution, or if they had received xenobiotic drugs (apart from routine anthelmintic or ectoparasiticidal treatment) or vaccines in the two week period prior to the onset of clinical signs.

Complete blood cell counts for dogs in the IMHA and INF groups were generated using the ADVIA 120 analyser (Siemens), and fresh blood smears were evaluated by a veterinary clinical pathologist in all cases. Serum biochemical profiles were produced with an ILAB 600 analyser (Instrumentation Laboratory).

Owners of all dogs were required to give informed consent for participation of their animal in the study, and the study had received approval from the Royal Veterinary College Clinical Research Ethical Review Board (reference number 2011_1134). Residual serum samples and residual samples of EDTA-anticoagulated blood were collected from the laboratory in which diagnostic samples had been analysed, though, in some cases, only one type of sample was available.

### Flow cytometry

Samples of EDTA-anticoagulated blood (volume 0.5 to 1.3 ml) were lysed using a buffer made by mixing 4.15 g ammonium chloride (VWR International), 18.5 mg disodium EDTA (SIGMA), and 0.5 g potassium bicarbonate (Sigma Aldrich) with 500 ml distilled water, adjusted to pH 7.4 and stored at 4°C. The blood sample was mixed with 10 ml of the lysis buffer and incubated at room temperature for seven minutes, then washed and re-suspended in phosphate-buffered saline (PBS) with 10% fetal calf serum (Biosera). The remaining peripheral blood mononuclear cells (PBMCs) were stained with fluorophore-conjugated antibodies specific for surface molecules (CD4, CD5 and CD8) and incubated at 4°C for 30 minutes, then washed and incubated in a fixation/permeabilisation buffer for 12 to 18 hours at 4°C in the dark, according to the manufacturers’ instructions (Foxp3/Transcription Factor Staining Buffer Set, Affymetrix eBioscience). The PBMCs were then stained with antibodies specific for intracellular molecules (CD79b and FoxP3). The antibody clones used in these experiments are shown in S1 Table. Cell staining was assessed by flow cytometry using a FACS Canto II cytometer (BD), and results were analysed using commercially available software (FlowJo, Treestar).

### Reverse transcription-polymerase chain reactions

Quantitative polymerase chain reactions were performed according to published guidelines [18]. Samples of EDTA-anticoagulated blood were added to a pre-mixed RNA stabilising solution (RiboPure RNA Purification kit—blood, ThermoFisher Scientific) and then stored at -80°C until further analysis was completed. Ribonucleic acid was extracted from PBMCs according to the manufacturer’s instructions, and genomic DNA was degraded using the DNase I provided. The concentration and purity of the RNA were determined by measurement of absorbance of light at wavelengths of 260 nm and 280 nm (NanoDrop ND-1000, Thermo Scientific).

Three reference genes were selected based on the stability of their expression in samples from dogs in the HC group (n = 5) and IMHA group (n = 5), as determined using a commercially available kit and associated software (geNorm Reference Gene Selection Kit with Double Dye (Hydrolysis) probe, with qBase+ software and 2x PrecisionPLUS Mastermix, Primerdesign). The reference genes selected were *b2m*, *sdha* and *rpl32*, with primers shown in S2 Table.

Relative expression of the genes of interest, *il10* and *ifng*, was determined by reverse transcriptase quantitative PCR, using the primers shown in S2 Table. All primers were obtained from Thermo Fisher Scientific. Each reaction well in a black 384 well plate (Bio-Rad) contained the primers (at a final concentration of 900 nM), fam probe (at 250 nM), a one-step reverse transcriptase and DNA polymerase enzyme solution (Precision Onestep qRT-PCR Mastermix, Primerdesign, 10 μl per well), and 25 ng of RNA in a total volume of 20 μl. The reaction plate was incubated at 55°C for 15 minutes to allow reverse transcription, before the DNA polymerase was activated by incubation at 95°C for 8 minutes. The plate then underwent 40 cycles of denaturation (95°C for 10 seconds) and extension with data collection (60°C for 60 seconds), all using a CFX 384 Real-Time PCR detection system (Bio-Rad).

Expression of each gene was assessed in duplicate for each dog. The relative expression of each gene was then quantified using Eq 1, where AE refers to the geometric mean of the amplification efficiency values for the reference genes and the gene of interest, and Cq refers to the cycle in which fluorescence was first detected. Amplification efficiency values were calculated from five point standard curves constructed for each set of primers using Eq 2.

$$Relative\;expression=\;{\left(AE\right)}^{\left(Cq\left[gene\;of\;interest\right]-Cq\left[geometric\;mean\;of\;reference\;genes\right]\right)}$$ (1)

$$Amplification\;Efficiency=\;10^{\left(-\frac{1}{slope}\right)}$$ (2)

### Cytokine measurements

Serum samples were collected and stored in plain polyethylene tubes (Greiner Bio-one) at -80°C until further analyses were completed. The serum concentrations of IL-2, 6 and 10, TNFα and CXCL8 were measured using enhanced chemiluminescent assays (Canine Proinflammatory Panel 3 Ultra-Sensitive Kit and Canine IL-10 Assay Ultra-Sensitive Kit, Meso Scale Discovery), as described previously in dogs [19]. Chemiluminescence was measured using a SECTOR S 600 instrument (Meso Scale Discovery). Cytokine measurements were made in duplicate for each dog, and individual values were calculated after fitting a standard curve using four-parameter logistic regression, with 1/Y^2^ weighting.

### Statistical analysis

Analyses were conducted using statistical software packages (SPSS version 21, IBM and GraphPad Prism version 6, GraphPad Software). Variables were assessed for normality by visual inspection of histograms and using the Shapiro-Wilks test. Non-parametric variables were compared using the Mann-Whitney U test and Kruskal-Wallis test, whereas normally distributed variables were compared with the Student’s t test. Categorical variables were compared using the Chi squared test or Fisher’s exact test, depending on the number of cases per cell.

The nature of the underlying variability in the set of immunological and haematological variables was explored by principal component analysis with varimax rotation, using INF (n = 10) and IMHA (n = 6) dogs that had a complete set of data available. Components were extracted if they had eigenvalues greater than 1; scores were calculated for each animal for each component by regression, and these were then compared between groups.

The manuscript was presented according to the STROBE statement for standardised reporting of observational studies [20]. All data obtained in this study are available in S1 File.

## Results

### Recruitment of cases

We collected samples from 32 dogs with IMHA, of which 12 were excluded because they had received immunosuppressive treatment prior to presentation to our institution. One further dog was excluded because it was suffering a relapse of the disease and had undergone splenectomy previously. The remaining dogs had a median age of 8.0 years (inter-quartile range [IQR]: 3.9–9.6) and included one entire male, 9 castrated males, one entire female and 8 neutered females. Ten different breeds were represented, of which the most frequent were cocker spaniels (n = 4), English Springer spaniels (n = 3), Labrador retrievers (n = 3), and cross-breed dogs (n = 4) (Table 1). Diagnosis of IMHA was based on confirmation of anaemia in all cases (median packed cell volume 15.0%, IQR: 10.5–17.5, range: 5.0–29.0) and the presence of spherocytes on a blood smear (n = 15, 78.9%), or continued agglutination after dilution in saline (n = 14, 73.7%), or a titre of ≥1:16 in the DAT (n = 2, 10.5%).

**Table 1:** Demographic characteristics of the three groups of dogs recruited to this study.

Group		IMHA^a^	INF^b^	HC^c^
**N**		19	22	31
**Age (years)**				
	Median	8.0	5	5.7
	IQR^d^	3.9–9.6	2.6–7.0	4.0–7.0
**Sex (N)**				
	Male entire	1	7	3
	Male neutered	9	5	10
	Female entire	1	3	1
	Female neutered	8	7	17
**Breed (N, four most frequent)**		Cocker spaniel (n = 4), English springer spaniel (3), cross breed (3), Labrador retriever (3)	Cross breed (n = 3), English springer spaniel (3), Dachshund (2), Labrador retriever (2)	Cross breed (n = 6), Labrador retriever (6), Staffordshire bull terrier (2), greyhound(2)

^a^: healthy control;

^b^: inflammatory disease;

^c^: immune-mediated haemolytic anaemia;

^d^: inter-quartile range.

Samples were also obtained from 26 dogs with inflammatory diseases, of which two were excluded because they were presumptively diagnosed with immune-mediated diseases (sterile lympadenitis in one dog and immune-mediated polyarthritis in the other), and two others were excluded because they were diagnosed with cancer (lymphoma in one dog and haemangiosarcoma in the other). The median age of the 22 dogs that were included was 5.0 years (IQR: 2.6–7.0), with sex distribution shown in Table 1. The final diagnoses for dogs in this group included acute onset diarrhoea and/or vomiting (n = 6), pancreatitis (4), aspiration pneumonia (2), leptospirosis (2), bacterial endocarditis (1), chronic hepatitis (1), idiopathic haemorrhagic diarrhoea syndrome (1), prostatitis (1), retrobulbar cellulitis (1), urinary tract infection (1), and oesophageal stricture (1).

Thirty-one healthy dogs were included in the study, for which the median age was 5.7 years (IQR: 4.0–7.0). There was no difference in age between the three groups (Kruskal-Wallis test, *p* = 0.174), nor in the proportion of male and female dogs (Pearson Chi squared, *p* = 0.498).

### Frequency of regulatory T cells was similar among groups

**Fig 1:**
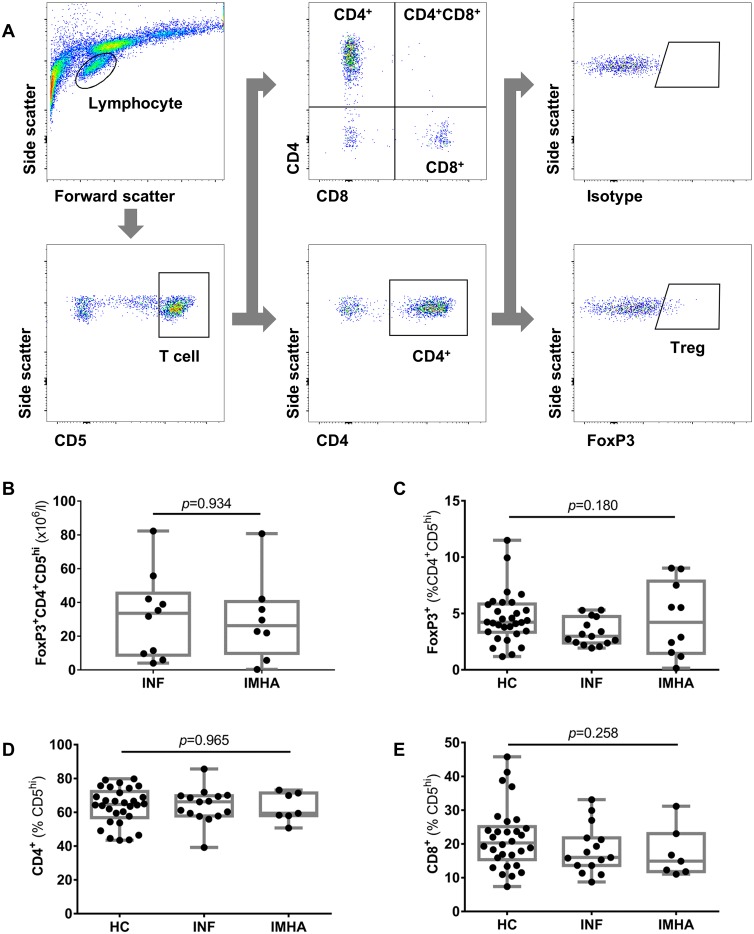
Flow cytometric characterisation of lymphocyte subsets. Peripheral blood samples underwent red blood cell lysis and the PBMCs were analysed by flow cytometry using the gating strategy shown (A). The proportions (B) and absolute numbers (C)of regulatory T cells, (D) CD4^+^ T cells and (E) CD8^+^ T cells were similar among the different groups of dogs. Groups were compared using Kruskal-Wallis tests. IMHA: immune-mediated haemolytic anaemia, INF: inflammatory disease, HC: healthy control.

To test our hypothesis that the frequency of regulatory T cells is reduced in dogs with IMHA compared to healthy dogs and those with inflammatory diseases, we performed flow cytometric analysis of PBMCs after lysis of red blood cells. We identified regulatory T cells using the cascaded gating strategy outlined in Fig 1A, where a population of lymphocytes was selected sequentially based on its expression of CD5, CD4 and FoxP3. The median frequency of FoxP3^+^ T cells (expressed as a percentage of the CD4^+^CD5^hi^ population and taken as Tregs) was 4.2% (IQR: 1.6–7.5, n = 10) in the IMHA group, 3.0% (IQR: 2.4–4.4, n = 15) in the INF group, and 4.2% (IQR: 3.4–5.8, n = 30) in healthy dogs, with no significant difference between groups (Kruskal-Wallis, *p* = 0.180). The frequencies of CD4^+^ T cells (*p* = 0.965) and CD8^+^ T cells (*p* = 0.258) were also similar among groups (Fig 1).

### IMHA was associated with increased production of pro-inflammatory cytokines

**Fig 2:**
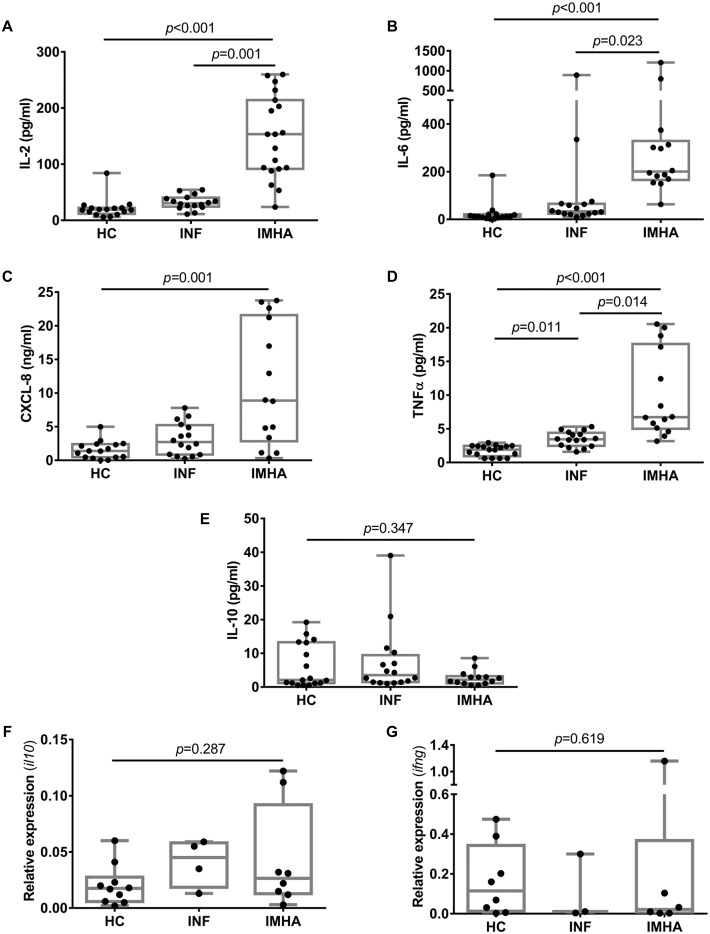
Cytokine balance in dogs with IMHA. Measurement of cytokines in peripheral blood indicated greater concentrations of pro-inflammatory cytokines in dogs with IMHA compared to healthy controls (A-D), with similar concentrations of IL-10 among all three groups (E). The relative expression of *il10* (encoding IL-10) (F) and *ifng* (encoding IFNγ) (G) was similar among groups. Groups were compared using Kruskal-Wallis tests, with *post-hoc* pairwise tests. IMHA: immune-mediated haemolytic anaemia, INF: inflammatory disease, HC: healthy control.

Next, we explored the hypothesis that development of IMHA in dogs is associated with an imbalance between cytokines that have largely pro- and anti-inflammatory effects. We found that cytokines with a pro-inflammatory effect (IL-2, IL-6, CXCL-8 and TNFα) were present in the serum of dogs with IMHA at significantly greater concentrations than in the HC group (Fig 2A–2D). Interestingly, the concentration of TNFα was greater in the IMHA group than INF group, and greater in the INF group than the HC group, suggesting that development of IMHA is associated with a stronger inflammatory response than in diseases that were not of autoimmune aetiology. Conversely, the concentration of IL-10, which usually exerts a broadly anti-inflammatory effect, was similar among the three groups (Fig 2E). We also examined the expression of cytokine genes in RNA extracted from PBMCs and found no difference in relative expression of the genes encoding IL-10 and IFNγ among the three groups (Kruskal-Wallis, *il10*: p = 0.287; *infg*: p = 0.619) (Fig 2F and 2G).

### Variance of immunological variables was largely attributable to four components

**Fig 3:**
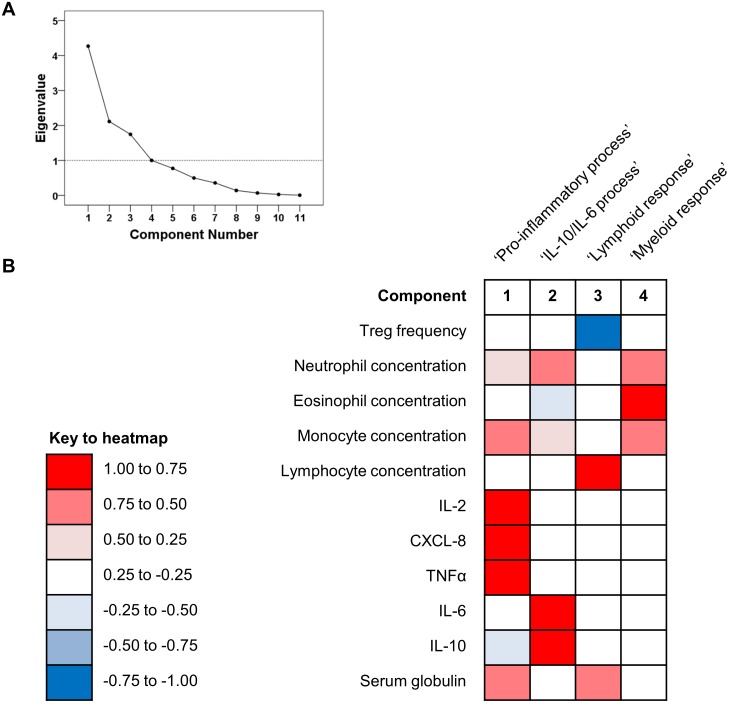
Results of principal component analysis (n = 16 dogs). Four variables explained the majority of the variability in the dataset, as indicated in the scree plot (A). The loading scores represented in the heatmap (B) indicate the strength of correlation, either positive or negative, between individual variables and the components extracted in this model. Strong positive (>0.5) or negative (< -0.5) correlations indicate that the variable contributed greatly to the component, whereas weaker correlations indicate that the variable made little contribution to the component.

We wished to determine whether a smaller number of underlying factors explained changes in the immunological and haematological variables measured in this study. We therefore conducted principal component analysis to explore the entire dataset further because this approach precluded multiple statistical tests to assess correlations between single variables. Using eleven input variables, we identified four major components, which collectively accounted for 83.01% of the variability in the dataset (Fig 3A). Based on their loading scores (Fig 3B, S3 Table), we named the four components ‘pro-inflammatory process’, which was highly correlated with the concentrations of the major pro-inflammatory cytokines but negatively correlated with IL-10; ‘IL-10/IL-6 process’; ‘lymphoid response’, associated with the lymphocyte and serum globulin concentrations; and ‘myeloid response’, characterised by strong correlations with the concentrations of granulocytes and monocytes. Assessment of the model revealed adequate sampling (Bartlett’s test of sphericity, *p*<0.001) and accuracy (Kaiser-Meyer-Olkin measure 0.388).

**Fig 4:**
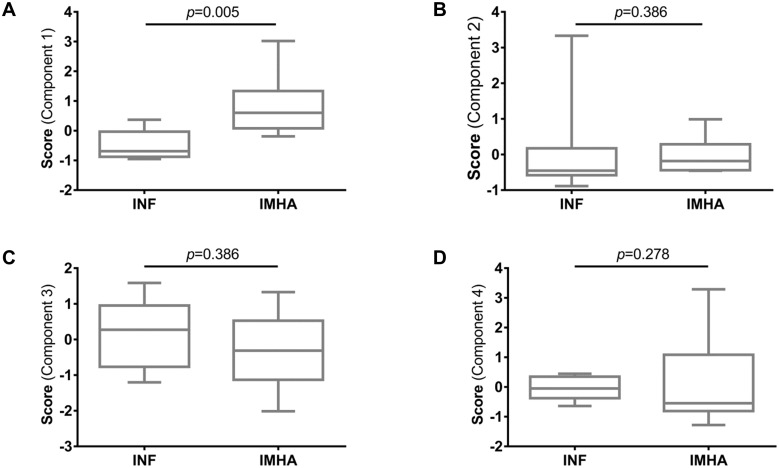
Comparison of individual scores for components: Individual scores were derived for each animal for each component. Scores for the component named the ‘pro-inflamamtory process’ were greater in dogs with IMHA than in those with inflammatory diseases (A), whereas the scores for the other components were similar between groups (B-D). Scores were compared with Mann-Whitney *U* tests. IMHA: immune-mediated haemolytic anaemia, INF: inflammatory disease.

We derived the scores for each component for each dog in the study and found that scores for component 1, the ‘pro-inflammatory process’, were significantly greater in dogs with IMHA (Fig 4A) whereas scores for the other components were similar between groups (Fig 4B–4D), confirming that the immunophenotype of dogs with IMHA was distinct from that of dogs with other inflammatory diseases.

## Discussion

In this study, we found that the frequency of Tregs in peripheral blood did not differ between dogs with primary IMHA and other groups, but we found that the concentrations of pro-inflammatory cytokines were greater in dogs with IMHA compared to other groups. After condensing the number of dimensions in the dataset by principal component analysis, we identified four major components that accounted for the majority of the variability among the immunological and haematological variables. Scores for one of these components (positively correlated with the concentrations of IL-2, CXCL-8, TNFα, neutrophils and monocytes, and negatively correlated with IL-10) differed significantly between dogs with IMHA and those with inflammatory diseases, but there was no difference in scores when comparing the other three components between groups.

The role of thymic Tregs in development and progression of autoimmune diseases is an area of intense investigation in experimental models and in human medicine (as reviewed by Buckner [21]). Presence of functioning Tregs is required to prevent development of autoimmune diseases, as demonstrated in mice that have undergone day 3 thymectomy, cannot express the Foxp3 transcription factor, or have undergone pharmacological depletion of Tregs after formation of a mature immune system [22–25]. Similarly, development of spontaneous autoimmune diseases is observed in people with mutations in the gene encoding FOXP3, resulting in a group of diseases described as immunodysregulation polyendocrinopathy enteropathy X-linked syndrome (IPEX) [26,27].

In contrast to the findings of our study, the frequency of thymic Tregs in peripheral blood of people with warm AIHA was lower than that of healthy volunteers [28] in a study that recruited a similar number of patients as our own investigation. Depletion of Tregs by administration of an anti-CD25 antibody also resulted in a faster onset and more severe form of disease in a murine model of IMHA induced by administration of rat erythrocytes [29]. Although numbers of Tregs in peripheral blood were unaltered in dogs with IMHA, it might have been more pertinent to investigate relative proportions of regulatory cells in the liver and spleen because the MPS of these organs is considered to be the principal site of destruction of red blood cells in affected animals [1]. This strategy has proven to be informative in studies of rheumatoid arthritis in people, in whom the frequency of Tregs is consistently increased in synovial fluid [30–32] but may be increased, normal or decreased in peripheral blood when compared to healthy controls (summarised by Miyara et al, 2011 [33]). To our knowledge, the lymphoid populations of the liver and spleen have yet to be studied in dogs with IMHA, which is likely to be related to the difficulty in obtaining such samples in living animals.

The functional properties of Tregs also were not evaluated in this study but this is an important area for future investigation because changes in suppressive activity could be associated with development of autoimmune disease, even without changes in the numbers of cells present [21]. This notion is supported by studies of people with type 1 diabetes mellitus and multiple sclerosis, in whom the suppressive activity of Tregs was impaired in assays performed *in vitro* [34,35].

We investigated the relative frequency and absolute numbers of CD4^+^FoxP3^+^ Tregs in this study because the phenotype and suppressive activity of this population of cells has been characterised extensively in dogs [7,8,36]. However, previous studies indicate that conventional T helper cells also may be induced to adopt a regulatory Tr1 phenotype in people with AIHA [37,38]. These Tr1 cells produced IL-10 in response to peptides derived from the immunodominant Rhesus D molecule, such that they were able to suppress production of IFNγ by PBMCs when cultured together. Preliminary investigations in our own laboratory suggest that Tr1 cells also may be induced in dogs, though the role of these cells in development of IMHA has not yet been investigated.

Production of cytokines was investigated using two methods in this study: by direct measurement of the serum concentrations of five cytokines and by quantitative PCR to evaluate expression of the genes encoding two cytokine genes. These approaches should provide complementary information by defining the nature of the cytokine milieu at the point of diagnosis and also indicating which cytokines are being produced by PBMCs at the same stage in the autoimmune response. The results of these assays indicated that development of IMHA was associated with high serum concentrations of cytokines that are generally considered to have pro-inflammatory effects, but it is not possible to determine whether these changes were contributing to the development of the disease or whether they occurred in response to the destruction of red blood cells.

An increased serum concentration of IL-2 has been reported previously in dogs with IMHA [39] and in people with AIHA [40], and there has been speculation that this abnormality might be associated with an increased tendency for T cells to proliferate *in vitro* in the latter group [41]. According to traditional models, increased concentrations of IL-2, CXCL-8 and TNFα suggested that the immune response might be driven by Th1 cells, resulting in activation of phagocytes and other cells capable of clearing intracellular pathogens [42]. The autoimmune response in patients with AIHA was previously considered to have a Th1 phenotype because PBMCs stimulated *in vitro* with self-antigens were found to produce large amounts of the signature cytokine IFNγ [37].

Since the discovery of the Th17 subset of helper T cells, it has become increasingly apparent that several autoimmune diseases are strongly associated with inappropriate Th17 activity [43]. In people with AIHA, increased frequencies of Th17 cells in peripheral blood and an increased serum concentration of IL-17 have been observed; the concentration of IL-17 was also correlated with disease activity [44,45]. Interleukin-17 is also able to cause activation of phagocytes, resulting in release of CXCL-8, IL-6 and TNFα [46]. This discussion raises the interesting possibility that IMHA in dogs also could be driven by an autoimmune response with a Th17 phenotype, resulting in phagocyte activation and production of pro-inflammatory cytokines. Further characterisation of Th17 responses will be an important area for future research in dogs with autoimmune diseases.

There was no difference in the serum concentration of IL-10 among groups, nor in the relative expression of the *il10* gene. These results differ from those reported in another study of IMHA in dogs [39] and from *in vitro* studies of PBMCs from people with wIMHA [28,47,48], in which the concentration of IL-10 was increased in affected individuals. Interleukin 10 is considered to be an important regulatory cytokine, yet serum concentrations appear to be paradoxically high in diseases characterised by production of autoantibodies, including AIHA and systemic lupus erythematosus (SLE) [49]. In the latter disease, increased IL-10 secretion permits survival of autoreactive B cells and encourages production of pathogenic antibodies [50].

We identified several individual dogs, even among those that were considered to be healthy controls, which had particularly high concentrations of some serum cytokines. It is possible that some of these dogs had occult inflammatory or immune-mediated diseases, but no diagnoses had been made by the time the study concluded. Production of these cytokines could also vary with genetic background, age, sex or neuter status in dogs, as has been reported in people [51–53]. In addition, inflammatory diseases that are strongly associated with particular breeds could develop due to abnormalities in the innate immune system, as has been described in Chinese Shar pei dogs with recurrent fevers caused by overproduction of IL-6 [54].

In an effort to explore our dataset for latent variables, we performed principal component analysis using haematological and immunological variables. Unsurprisingly, the predominant component was positively correlated with the pro-inflammatory cytokines (IL-2, CXCL-8 and TNFα) but negatively correlated with the serum concertation of IL-10, and scores for this component differed between groups.

A second but separate component was positively correlated with the serum concentrations of IL-6 and IL-10, which recalls an interesting notion proposed by other workers that some autoimmune responses may be driven by an imbalance in the concentrations of these two cytokines [55]. In particular, studies of mice with experimental autoimmune encephalitis (a model of human multiple sclerosis) indicated that excessive concentrations of IL-6 interfere with the action of IL-10, inhibiting the regulatory action of this cytokine on autoreactive B cells and other components of the innate and adaptive immune system [56]. Interestingly, we also found that the concentration of IL-6 was significant greater in dogs with IMHA compared to either healthy controls or dogs with inflammatory diseases, which finding contradicts a previous study which did not demonstrate a difference in the concentration of this cytokine in dogs with IMHA compared to healthy controls [39]. In that study, the concentration of IL-6 was below the lower limit of detection in all healthy controls and in at least half of the dogs with IMHA, which led the authors to speculate that failure to observe the expected difference could have been related to poor analytical sensitivity.

The final two components appeared to correspond to different types of inflammatory response, with a third component positively correlated with the lymphocyte count and serum globulin concentration. This could reflect increased production of immunoglobulin by lymphocytes in chronic inflammatory states, even with normal total concentrations of serum globulin, but we did not undertake serum protein immunoelectrophoresis to test this hypothesis. The final component was highly correlated with the concentrations of the major leukocytes, particularly those of the granulocytes and monocytes. We described this component as a ‘myeloid response’, and leukocytosis has been noted in a large proportion of dogs and identified as a negative prognostic indicator in previous studies of IMHA [57–59]. We suspect that this response is driven by the production of pro-inflammatory cytokines, which are likely to affect haemopoietic stem cells directly [60], and to result in the extramedullary haemopoiesis that we have observed often in the livers and spleens of affected dogs.

The difference in the score for the ‘pro-inflammatory process’ between groups highlights the difference between dogs with IMHA and those included in the inflammatory disease group, which acted as a positive control group for this study. The difference in concentration of TNFα among groups indicated that development of IMHA results in a stronger pro-inflammatory response than the various inflammatory diseases though we cannot determine, based on this study, whether this is a direct result of the autoimmune response or related to the host response to release of damage-associated molecular patterns from disrupted red blood cells.

We chose to conduct principal component analysis to allow us to integrate the data from multiple parameters and to explore the dataset in a way that would facilitate future investigations. Whereas this approach avoided multiple individual statistical comparisons and associated risk of errors, our analysis may have been limited by the small number of individuals included and the relatively high case-to-variable ratio. Furthermore, the components identified should not be considered causative factors in the development of disease but rather as statistical constructs that help to explain the results obtained in this investigation.

This study has a number of additional limitations, largely related to the relatively small number of cases, whichmay have reduced the power to detect small differences between groups. With a small number of exceptions, all samples were collected at the point of presentation of diseased dogs to the study institution. Because the autoimmune response had already progressed to the point of causing clinical disease by this time and because follow-up samples were not available, causality could not be ascribed in this study, and the effects of treatment on the autoimmune response could not be determined. Our study omitted some important factors that would have helped us to characterise the autoimmune response more completely, such as the serum concentrations of IL-17 and IFNγ and the frequencies of Th1 and Th17 T cells, though these are areas of planned investigation.

In conclusion, we found that dogs with IMHA had greater serum concentrations of pro-inflammatory cytokines than dogs with inflammatory diseases, but there was no difference in the frequency of Tregs or the regulatory cytokine IL-10. Further investigations will be required to define the character of the autoimmune response and elucidate possible therapeutic targets, and to determine whether these immunological changes have any impact on survival.

## Supporting Information

S1 Table Antibody clones used for flow cytometry. (DOCX)

S2 Table Details of primers used for reverse transcription polymerase chain reactions. All primers were obtained from ThermoFisher Scientific.
(DOCX)

S3 Table Loading of variables onto components extracted from principal component analysis (n = 16 dogs). Loading scores indicate the strength of correlation, either positive or negative, between individual variables and the components extracted in this model, and these correlations are also represented using the colours shown in the key. Strong positive (>0.5) or negative (< -0.5) correlations indicate that the variable contributed greatly to the component, whereas weaker correlations indicate that the variable made little contribution to the component.
(DOCX)

S1 File Spreadsheet of data obtained in this study. (XLSX)

## References

[1] McCullough S. Immune-mediated hemolytic anemia: understanding the nemesis. Vet Clin North Am Small Anim Pract. 2003;33: 1295–1315. 14664200 10.1016/j.cvsm.2003.08.003

[2] Berentsen S, Sundic T. Red blood cell destruction in autoimmune hemolytic anemia: role of complement and potential new targets for therapy. Biomed Res Int. 2015;2015:363278 10.1155/2015/363278 25705656 PMC4326213

[3] Barker RN, Gruffydd-Jones TJ, Stokes CR, Elson CJ. Identification of autoantigens in canine autoimmune haemolytic anaemia. Clin Exp Immunol. 1991;85: 33–40. 2070560 10.1111/j.1365-2249.1991.tb05678.x PMC1535729

[4] Corato A, Shen CR, Mazza G, Barker RN, Day MJ. Proliferative responses of peripheral blood mononuclear cells from normal dogs and dogs with autoimmune haemolytic anaemia to red blood cell antigens. Vet Immunol Immunopathol. 1997;59: 191–204. 9477471 10.1016/s0165-2427(97)00032-9

[5] Giltiay NV, Chappell CP, Clark EA. B-cell selection and the development of autoantibodies. Arthritis Res Ther. 2012;14 Suppl 4:S1. 10.1186/ar3918 PMC3535718 23281837

[6] Sakaguchi S, Wing K, Onishi Y, Prieto-Martin P, Yamaguchi T. Regulatory T cells: how do they suppress immune responses? Int Immunol. 2009;21: 1105–1111. 10.1093/intimm/dxp095 19737784

[7] Garden OA, Pinheiro D, Cunningham F. All creatures great and small: regulatory T cells in mice, humans, dogs and other domestic animal species. Int Immunopharmacol. 2011;11: 576–588. 10.1016/j.intimp.2010.11.003 21093606

[8] Pinheiro D, Singh Y, Grant CR, Appleton RC, Sacchini F, Walker KR, et al Phenotypic and functional characterization of a CD4(+) CD25(high) FOXP3(high) regulatory T-cell population in the dog. Immunology. 2011;132: 111–122. 10.1111/j.1365-2567.2010.03346.x 20880379 PMC3015081

[9] Wing K, Onishi Y, Prieto-Martin P, Yamaguchi T, Miyara M, Fehervari Z, et al CTLA-4 control over Foxp3+ regulatory T cell function. Science. 2008;322: 271–275. 10.1126/science.1160062 18845758

[10] Doganci A, Eigenbrod T, Krug N, De Sanctis GT, Hausding M, Erpenbeck VJ, et al The IL-6R alpha chain controls lung CD4+CD25+ Treg development and function during allergic airway inflammation in vivo. J Clin Invest. 2005;115: 313–325. 10.1172/JCI22433 15668741 PMC544603

[11] Valencia X, Stephens G, Goldbach-Mansky R, Wilson M, Shevach EM, Lipsky PE. TNF downmodulates the function of human CD4+CD25hi T-regulatory cells. Blood. 2006;108: 253–261. 10.1182/blood-2005-11-4567 16537805 PMC1895836

[12] Willerford DM, Chen J, Ferry JA, Davidson L, Ma A, Alt FW. Interleukin-2 receptor alpha chain regulates the size and content of the peripheral lymphoid compartment. Immunity. 1995;3: 521–530. 7584142 10.1016/1074-7613(95)90180-9

[13] Murai M, Turovskaya O, Kim G, Madan R, Karp CL, Cheroutre H, et al Interleukin 10 acts on regulatory T cells to maintain expression of the transcription factor Foxp3 and suppressive function in mice with colitis. Nat Immunol. 2009;10: 1178–1184. 10.1038/ni.1791 19783988 PMC2898179

[14] Sadlack B, Merz H, Schorle H, Schimpl A, Feller AC, Horak I. Ulcerative colitis-like disease in mice with a disrupted interleukin-2 gene. Cell. 1993;75: 253–261. 8402910 10.1016/0092-8674(93)80067-o

[15] Davidson NJ, Fort MM, Muller W, Leach MW, Rennick DM. Chronic colitis in IL-10-/- mice: insufficient counter regulation of a Th1 response. Int Rev Immunol. 2000;19: 91–121. 10723680 10.3109/08830180009048392

[16] Swann JW, Garden OA. Novel immunotherapies for immune-mediated haemolytic anaemia in dogs and people. Vet J. 2016;207: 13–19. 27152382 10.1016/j.tvjl.2015.10.022

[17] Zanella A, Barcellini W. Treatment of autoimmune hemolytic anemias. Haematologica. 2014;99: 1547–1554. 10.3324/haematol.2014.114561 25271314 PMC4181250

[18] Bustin SA, Benes V, Garson JA, Hellemans J, Huggett J, Kubista M, et al The MIQE guidelines: minimum information for publication of quantitative real-time PCR experiments. Clin Chem. 2009; 55: 611–622. 10.1373/clinchem.2008.112797 19246619

[19] Titmarsh HF, Gow AG, Kilpatrick S, Cartwright JA, Milne EM, Philbey AW, et al Low Vitamin D Status Is Associated with Systemic and Gastrointestinal Inflammation in Dogs with a Chronic Enteropathy. PloS one. 2015;10:e0137377 10.1371/journal.pone.0137377 26333093 PMC4557950

[20] von Elm E, Altman DG, Egger M, Pocock SJ, Gotzsche PC, Vandenbroucke JP, et al The Strengthening the Reporting of Observational Studies in Epidemiology (STROBE) statement: guidelines for reporting observational studies. J Clin Epidemiol. 2008;61: 344–349. 10.1016/j.jclinepi.2007.11.008 18313558

[21] Buckner JH. Mechanisms of impaired regulation by CD4(+)CD25(+)FOXP3(+) regulatory T cells in human autoimmune diseases. Nat Rev Immunol. 2010;10: 849–859. 10.1038/nri2889 21107346 PMC3046807

[22] Kojima A, Prehn RT. Genetic susceptibility to post-thymectomy autoimmune diseases in mice. Immunogenetics. 1981;14: 15–27. 7035348 10.1007/BF00344296

[23] Brunkow ME, Jeffery EW, Hjerrild KA, Paeper B, Clark LB, Yasayko SA, et al Disruption of a new forkhead/winged-helix protein, scurfin, results in the fatal lymphoproliferative disorder of the scurfy mouse. Nat Genet. 2001;27: 68–73. 11138001 10.1038/83784

[24] Kim JM, Rasmussen JP, Rudensky AY. Regulatory T cells prevent catastrophic autoimmunity throughout the lifespan of mice. Nat Immunol. 2007;8: 191–197. 17136045 10.1038/ni1428

[25] Ellis JS, Wan X, Braley-Mullen H. Transient depletion of CD4+ CD25+ regulatory T cells results in multiple autoimmune diseases in wild-type and B-cell-deficient NOD mice. Immunology. 2013;139: 179–186. 10.1111/imm.12065 23293979 PMC3647184

[26] Bennett CL, Christie J, Ramsdell F, Brunkow ME, Ferguson PJ, Whitesell L, et al The immune dysregulation, polyendocrinopathy, enteropathy, X-linked syndrome (IPEX) is caused by mutations of FOXP3. Nat Genet. 2001;27: 20–21. 11137993 10.1038/83713

[27] Wildin RS, Ramsdell F, Peake J, Faravelli F, Casanova JL, Buist N, et al X-linked neonatal diabetes mellitus, enteropathy and endocrinopathy syndrome is the human equivalent of mouse scurfy. Nat Genet. 2001;27: 18–20. 11137992 10.1038/83707

[28] Ahmad E, Elgohary T, Ibrahim H. Naturally occurring regulatory T cells and interleukins 10 and 12 in the pathogenesis of idiopathic warm autoimmune hemolytic anemia. J Investig Allergol Clin Immunol. 2011;21: 297–304. 21721376

[29] Mqadmi A, Zheng X, Yazdanbakhsh K. CD4+CD25+ regulatory T cells control induction of autoimmune hemolytic anemia. Blood. 2005;105: 3746–3748. 10.1182/blood-2004-12-4692 15637139 PMC1895013

[30] Cao D, van Vollenhoven R, Klareskog L, Trollmo C, Malmstrom V. CD25brightCD4+ regulatory T cells are enriched in inflamed joints of patients with chronic rheumatic disease. Arthritis Res Ther. 2004;6: R335–346. 10.1186/ar1192 15225369 PMC464877

[31] Lawson CA, Brown AK, Bejarano V, Douglas SH, Burgoyne CH, Greenstein AS, et al Early rheumatoid arthritis is associated with a deficit in the CD4+CD25high regulatory T cell population in peripheral blood. Rheumatology. 2006;45: 1210–1217. 10.1093/rheumatology/kel089 16571607

[32] Jiao Z, Wang W, Jia R, Li J, You H, Chen L, et al Accumulation of FoxP3-expressing CD4+CD25+ T cells with distinct chemokine receptors in synovial fluid of patients with active rheumatoid arthritis. Scand J Rheumatol. 2007;36: 428–433. 10.1080/03009740701482800 18092263

[33] Miyara M, Gorochov G, Ehrenstein M, Musset L, Sakaguchi S, Amoura Z. Human FoxP3+ regulatory T cells in systemic autoimmune diseases. Autoimmun Rev. 2011;10: 744–755. 10.1016/j.autrev.2011.05.004 21621000

[34] Viglietta V, Baecher-Allan C, Weiner HL, Hafler DA. Loss of functional suppression by CD4+CD25+ regulatory T cells in patients with multiple sclerosis. J Exp Med. 2004;199: 971–979. 10.1084/jem.20031579 15067033 PMC2211881

[35] Lindley S, Dayan CM, Bishop A, Roep BO, Peakman M, Tree TI. Defective suppressor function in CD4(+)CD25(+) T-cells from patients with type 1 diabetes. Diabetes. 2005;54: 92–99. 15616015 10.2337/diabetes.54.1.92

[36] Knueppel A, Lange S, Sekora A, Altmann S, Freund M, Junghanss C. Phenotypic and functional characterization of freshly isolated and expanded canine regulatory T cells. Exp Anim. 2011;60: 471–479. 22041284 10.1538/expanim.60.471

[37] Hall AM, Ward FJ, Vickers MA, Stott LM, Urbaniak SJ, Barker RN. Interleukin-10-mediated regulatory T-cell responses to epitopes on a human red blood cell autoantigen. Blood. 2002;100: 4529–4536. 10.1182/blood-2002-05-1383 12393426

[38] Ward FJ, Hall AM, Cairns LS, Leggat AS, Urbaniak SJ, Vickers MA, et al Clonal regulatory T cells specific for a red blood cell autoantigen in human autoimmune hemolytic anemia. Blood. 2008;111: 680–687. 10.1182/blood-2007-07-101345 17761830 PMC2575838

[39] Kjelgaard-Hansen M, Goggs R, Wiinberg B, Chan DL. Use of serum concentrations of interleukin-18 and monocyte chemoattractant protein-1 as prognostic indicators in primary immune-mediated hemolytic anemia in dogs. J Vet Intern Med. 2011;25: 76–82. 10.1111/j.1939-1676.2010.0642.x 21092010

[40] Fagiolo E, Vigevani F, Pozzetto U. High cytokine serum levels in patients with autoimmune hemolytic anemia (AIHA). Immunol Invest. 1994;23: 449–456. 7851962 10.3109/08820139409066839

[41] Fagiolo E, Abenante L. Lymphocyte activation and cytokine production in autoimmune hemolytic anaemia (AIHA). Autoimmunity. 1996;24(3):147–156. 9020407 10.3109/08916939608995360

[42] Elson CJ, Barker RN. Helper T cells in antibody-mediated, organ-specific autoimmunity. Curr Opin Immunol. 2000;12: 664–669. 11102770 10.1016/s0952-7915(00)00160-6

[43] Han L, Yang J, Wang X, Li D, Lv L, Li B. Th17 cells in autoimmune diseases. Front Med. 2015;9: 10–19. 10.1007/s11684-015-0388-9 25652649

[44] Hall AM, Zamzami OM, Whibley N, Hampsey DP, Haggart AM, Vickers MA, et al Production of the effector cytokine interleukin-17, rather than interferon-gamma, is more strongly associated with autoimmune hemolytic anemia. Haematologica. 2012;97: 1494–1500. 10.3324/haematol.2011.060822 22419580 PMC3487588

[45] Xu L, Zhang T, Liu Z, Li Q, Xu Z, Ren T. Critical role of Th17 cells in development of autoimmune hemolytic anemia. Exp Hematol. 2012;40: 994–1004 10.1016/j.exphem.2012.08.008 22960264

[46] Aggarwal S, Gurney AL. IL-17: prototype member of an emerging cytokine family. J Leukoc Biol. 2002;71: 1–8. 11781375

[47] Fagiolo E, Terenzi CT. Enhanced IL-10 production in vitro by monocytes in autoimmune haemolytic anaemia. Immunol Invest. 1999;28: 347–352. 10574632 10.3109/08820139909062268

[48] Fagiolo E, Toriani-Terenzi C. Th1 and Th2 cytokine modulation by IL-10/IL-12 imbalance in autoimmune haemolytic anaemia (AIHA). Autoimmunity. 2002;35: 39–44. 11908705 10.1080/08916930290005891

[49] Llorente L, Richaud-Patin Y, Wijdenes J, Alcocer-Varela J, Maillot MC, Durand-Gasselin I, et al Spontaneous production of interleukin-10 by B lymphocytes and monocytes in systemic lupus erythematosus. Eur Cytokine Netw. 1993;4: 421–427. 8186374

[50] Llorente L, Zou W, Levy Y, Richaud-Patin Y, Wijdenes J, Alcocer-Varela J, et al Role of interleukin 10 in the B lymphocyte hyperactivity and autoantibody production of human systemic lupus erythematosus. J Exp Med. 1995;181: 839–844. 7869046 10.1084/jem.181.3.839 PMC2191898

[51] Pietschmann P, Gollob E, Brosch S, Hahn P, Kudlacek S, Willheim M, et al The effect of age and gender on cytokine production by human peripheral blood mononuclear cells and markers of bone metabolism. Exp Gerontol. 2003;38: 1119–1127. 14580865 10.1016/s0531-5565(03)00189-x

[52] Miller MA, Cappuccio FP. Ethnicity and inflammatory pathways—implications for vascular disease, vascular risk and therapeutic intervention. Curr Med Chem. 2007;14: 1409–1425. 17584053 10.2174/092986707780831131

[53] Kleiner G, Marcuzzi A, Zanin V, Monasta L, Zauli G. Cytokine levels in the serum of healthy subjects. Mediators Inflamm. 2013;2013:434010 10.1155/2013/434010 23533306 PMC3606775

[54] Rivas AL, Tintle L, Kimball ES, Scarlett J, Quimby FW. A canine febrile disorder associated with elevated interleukin-6. Clin Immunol Immunopathol. 1992;64: 36–45. 1606750 10.1016/0090-1229(92)90057-u

[55] Ireland SJ, Monson NL, Davis LS. Seeking balance: Potentiation and inhibition of multiple sclerosis autoimmune responses by IL-6 and IL-10. Cytokine. 2015;73: 236–244. 10.1016/j.cyto.2015.01.009 25794663 PMC4437890

[56] Saxena A, Khosraviani S, Noel S, Mohan D, Donner T, Hamad AR. Interleukin-10 paradox: A potent immunoregulatory cytokine that has been difficult to harness for immunotherapy. Cytokine. 2015;74:27–34. 10.1016/j.cyto.2014.10.031 25481648 PMC4454631

[57] McManus PM, Craig LE. Correlation between leukocytosis and necropsy findings in dogs with immune-mediated hemolytic anemia: 34 cases (1994–1999). J Am Vet Med Assoc. 2001;218: 1308–1313. 11330619 10.2460/javma.2001.218.1308

[58] Mitchell KD, Kruth SA, Wood RD, Jefferson B. Serum acute phase protein concentrations in dogs with autoimmune hemolytic anemia. J Vet Intern Med. 2009;23: 585–591. 10.1111/j.1939-1676.2009.0282.x 19298608

[59] Piek CJ, Brinkhof B, Teske E, Rothuizen J, Dekker A, Penning LC. High intravascular tissue factor expression in dogs with idiopathic immune-mediated haemolytic anaemia. Vet Immunol Immunopathol. 2011;144: 346–354. 10.1016/j.vetimm.2011.08.010 21899896

[60] King KY, Goodell MA. Inflammatory modulation of HSCs: viewing the HSC as a foundation for the immune response. Nat Rev Immunol. 2011;11: 685–692. 10.1038/nri3062 21904387 PMC4154310

