# Supplementary material for: Characterisation of the Immunophenotype of Dogs with Primary Immune-Mediated Haemolytic Anaemia
Source: PLoS One. 2016 Dec 12;11(12):e0168296. doi: 10.1371/journal.pone.0168296 (PMC5152924; doi:10.1371/journal.pone.0168296)
Supplement: S1 Table — (DOCX) [file pone.0168296.s001.docx]

S1 Table: Antibody clones used for flow cytometry.

| Marker | Fluorophore | Clone | Manufacturer | Species specificity |
| --- | --- | --- | --- | --- |
| CD4 | PE-Cy7 | YKIX302.9 | Affymetrix eBioscience | Dog |
| CD5 | R-PE^a^ | MCA1037PE | Bio-Rad | Dog |
| CD8a | eFluor 450 | YCATE55.9 | Affymetrix eBioscience | Dog |
| CD79b | FITC^b^ | MCA2209F | AbD Serotec | Mouse, cross-reactive to dog |
| FoxP3 | APC^c^ | FJK-16s | Affymetrix eBioscience | Mouse, cross-reactive to dog |

^a^: phycoerythrin

^b^: fluorescein isothiocyanate

^c^: allophycocyanin
