# Supplementary material for: Characterisation of the Immunophenotype of Dogs with Primary Immune-Mediated Haemolytic Anaemia
Source: PLoS One. 2016 Dec 12;11(12):e0168296. doi: 10.1371/journal.pone.0168296 (PMC5152924; doi:10.1371/journal.pone.0168296)
Supplement: S2 Table — All primers were obtained from ThermoFisher Scientific. (DOCX) [file pone.0168296.s002.docx]

S2 Table: Details of primers used for reverse transcription polymerase chain reactions, all from ThermoFisher Scientific.

| Gene | Encoded protein | NCBI reference sequence | Location on gene | Location | Amplicon length |
| --- | --- | --- | --- | --- | --- |
| *b2m* | Beta 2 microglobulin | XM_845055.1 | 107 | Exon 1-2 boundary | 87 |
| *sdha* | Succinate dehydrogenase complex (subunit A) | XM_535807.2 | 349 | Exon 3-4 boundary | 64 |
| *rpl32* | Ribosomal protein L32 | XM_854019.1 | 131 | Exon 1-2 boundary | 79 |
| *il10* | Interleukin-10 | XM_850467.1 | 444 | Exon 4-5 boundary | 64 |
| *ifng* | Interferon γ | NM_001003174.1 | 472 | Exon 3-4 boundary | 57 |
