# Supplementary material for: Characterisation of the Immunophenotype of Dogs with Primary Immune-Mediated Haemolytic Anaemia
Source: PLoS One. 2016 Dec 12;11(12):e0168296. doi: 10.1371/journal.pone.0168296 (PMC5152924; doi:10.1371/journal.pone.0168296)
Supplement: S3 Table — Loading scores indicate the strength of correlation, either positive or negative, between individual variables and the components extracted in this model, and these correlations are also represented using the colours shown in the key. Strong positive (>0.5) or negative (< -0.5) correlations indicate that the variable contributed greatly to the component, whereas weaker correlations indicate that the variable made little contribution to the component. (DOCX) [file pone.0168296.s003.docx]

**S3 Table: Loading of variables onto components extracted from principal component analysis (n=16 dogs).** Loading scores indicate the strength of correlation, either positive or negative, between individual variables and the components extracted in this model, and these correlations are also represented using the colours shown in the key. Strong positive (>0.5) or negative (< -0.5) correlations indicate that the variable contributed greatly to the component, whereas weaker correlations indicate that the variable made little contribution to the component.

| **Component** | **1** | **2** | **3** | **4** |
| --- | --- | --- | --- | --- |
| **Description** | ‘Pro-inflammatory process’ | ‘IL-10/IL-6 process’ | ‘Lymphoid response’ | ‘Myeloid response’ |
| **Variance (%)** | 34.83 | 16.76 | 16.35 | 15.06 |
| **Eigenvalue** | 3.831 | 1.844 | 1.799 | 1.657 |
|  |  |  |  |  |
| **Loading variable** |  |  |  |  |
| **Tregs (% CD4^+^CD5^+^ lymphocytes)** | 0.081 | 0.069 | -0.851 | 0.025 |
| **Neutrophil concentration (x10^9^/l)** | 0.383 | 0.516 | 0.153 | 0.698 |
| **Eosinophil concentration (x10^9^/l)** | -0.047 | -0.327 | 0.152 | 0.839 |
| **Monocyte concentration (x10^9^/l)** | 0.709 | 0.327 | -0.107 | 0.565 |
| **Lymphocyte concentration (x10^9^/l)** | -0.212 | -0.010 | 0.797 | 0.143 |
| **IL-2 (pg/ml)** | 0.940 | -0.054 | -0.095 | -0.034 |
| **CXCL-8 (pg/ml)** | 0.937 | 0.034 | -0.081 | 0.219 |
| **TNFα (pg/ml)** | 0.959 | -0.009 | -0.130 | -0.019 |
| **IL-6 (pg/ml)** | 0.245 | 0.876 | -0.237 | 0.025 |
| **IL-10 (pg/ml)** | -0.337 | 0.766 | 0.094 | -0.142 |
| **Serum globulin concentration (g/l)** | 0.524 | -0.044 | 0.533 | 0.233 |

Key to heat map:

|  |  |  |  |  |  |  |
| --- | --- | --- | --- | --- | --- | --- |
| -1.0 to -0.75 | -0.75 to -0.5 | -0.5 to -0.25 | -0.25 to 0.25 | 0.25-0.5 | 0.5-0.75 | 0.75-1.0 |
